# Cardiac Markers in Pediatric Laboratory Medicine: Critical Review

**DOI:** 10.3390/diagnostics15020165

**Published:** 2025-01-13

**Authors:** Renata Zrinski Topic, Jasna Lenicek Krleza

**Affiliations:** 1 Department of Laboratory Diagnostics, Children’s Hospital Zagreb, 10000 Zagreb, Croatia; renata.zrinski-topic@zg.t-com.hr; 2 Department of Laboratory Medical Diagnostics, University of Applied Health Sciences, 10000 Zagreb, Croatia; 3 Department of Nursing, Catholic University of Croatia, 10000 Zagreb, Croatia

**Keywords:** cardiac biomarker, cardiotoxicity, diagnosis, heart disease, natriuretic peptide, pediatrics, troponin

## Abstract

Currently, there are no validated guidelines or recommendations for how to interpret cardiac biomarkers in the pediatric population. The most commonly used cardiac biomarkers are cardiac troponins and natriuretic peptides, but the clinical value of common cardiac biomarkers in pediatric laboratory medicine is restricted due to age- and sex-specific interpretations, and there are no standardized cut-off values. The results from the studies on reference values, as well as results from clinical studies, are difficult to compare with identical studies due to the heterogeneity of subject characteristics (gestational and chronological age, sex, pubertal status, menstrual cycle, exercise), assay characteristics (type of assay, generation of assay, analytical platform used), and experimental protocol characteristics (prospective or retrospective studies, reference population selection, patient population selection, inclusion and exclusion criteria, number of subjects). Future studies need to establish evidence-based cut-offs for specific indications to optimize utilization and standardize the interpretation of common cardiac biomarkers in neonates, children, and adolescents. The aim of this article was to summarize the current analytical and clinical limitations of cardiac troponins and natriuretic peptides in the pediatric population, as informed by the existing published literature.

## 1. Introduction

Children are not small adults, and children’s hearts are not small hearts of adult persons. Therefore, pediatric cardiology includes a wide spectrum of etiologies and clinical manifestations that are very distinct from adult population cardiology. There are complex anatomical, physiological, and biochemical changes in the heart and cardiovascular system adaptation during the transition from fetal to neonatal life, as well as changes later during childhood and puberty.

**Figure 1:**
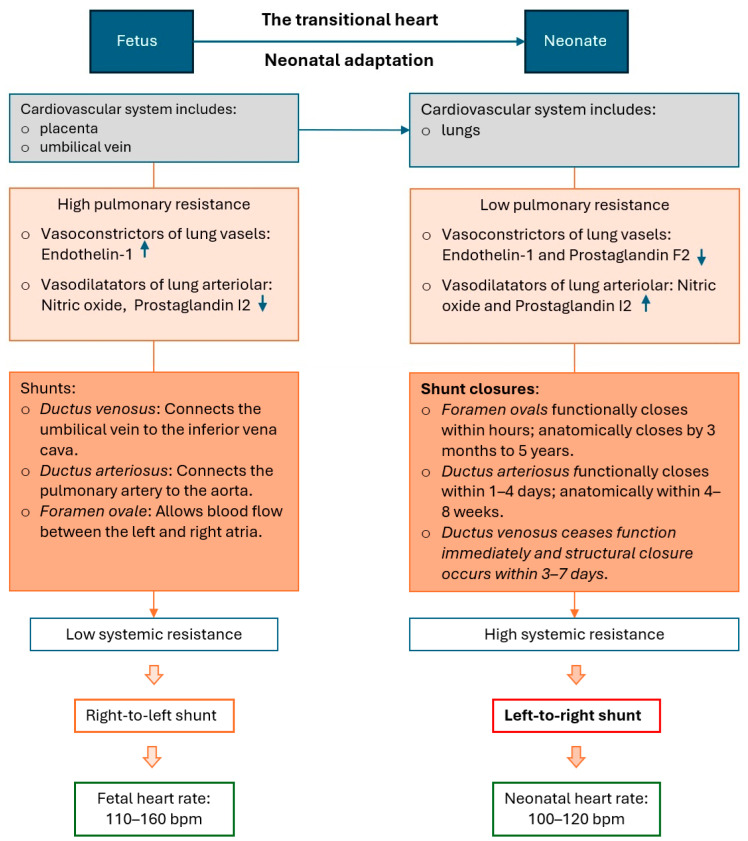
Transitional heart: adaptation of the fetal heart and circulation to neonatal life immediately after birth.

Fetus possesses structural and functional cardiovascular adaptations in the intrauterine environment. The transition from intrauterine to extrauterine life requires successful cardiopulmonary and cardiovascular remodeling in a timely manner to allow normal and independent life (Figure 1). However, many pregnancy complications, such as intrauterine growth restriction, fetal complications, placental dysfunction, preeclampsia, or preterm birth, can affect cardiac remodeling and transitional physiology and lead to long-term cardiovascular complications and diseases. In addition, heart disease in adults is predominantly acquired, whereas in children, it is primarily congenital, so pediatric cardiovascular diseases are not simply a replica of cardiovascular diseases seen in adult patients [1,2].

Heart disease mechanisms, clinical presentations, and frequency of heart diseases in children are quite discrepant from adults. Although the overall prevalence of cardiovascular disease in children is significantly lower compared with the adult population, it contributes significantly to pediatric mortality [1,2]. Pediatric heart diseases include congenital heart defects, cardiomyopathies, and myocarditis, with an increasing prevalence of atherosclerosis due to obesity and sedentary lifestyles [3]. Although recommended, physical activity also becomes a trigger for major adverse cardiovascular events, such as arrhythmias, sudden cardiac arrest, and sudden cardiac death in apparently healthy young athletes [4]. Cardiovascular complications are also linked to COVID-19 [5] and cancer therapies [6,7]. Key insights into pediatric heart diseases are presented in Table 1.

Circulating cardiac biomarkers are integral to the diagnosis, prognosis, and monitoring of cardiovascular diseases in patients with both primary and acquired heart conditions. The characteristics of cardiac biomarkers used in the diagnosis, prognosis, and monitoring of cardiac damage in adult patients are shown in Table 2 [17,18,19,20,21,22].

Cardiac troponins (cTn), B-type natriuretic peptide (BNP), and N-terminal pro-B-type natriuretic peptide (NT-proBNP) are firmly established as the leading biomarkers in clinical practice and current literature. It is essential to recognize that other cardiac biomarkers also provide significant insights into myocardial injury and heart failure (HF) in adults. Previously, before the introduction and clinical adoption of high-sensitivity cardiac troponin (hs-cTn) assays, creatine kinase-MB (CK-MB) and myoglobin were the main tools for diagnosing myocardial infarction (MI) in adults. However, given the superior sensitivity and specificity of cTn over CK-MB, myoglobin, LD, and AST, the Fourth Universal Definition of MI categorically recommends these older markers only when cTn is unavailable. This shift has led to many clinical laboratories discontinuing these assays. Therefore, it is clear that this review will not consider their use in pediatric cases [18,23].

However, the clinical value of common cardiac biomarkers in pediatric laboratory medicine is restricted due to age- and sex-specific interpretation, and there are no standardized cut-off values. In adult patients, cardiac biomarkers are recommended in the diagnosis of heart diseases, while the clinical values of cardiac biomarkers in pediatric patients require special consideration.

Despite these interpretative limitations, there is increasing literature to support their application in the diagnosis, differential diagnosis, monitoring, and prognosis of cardiovascular disease in neonates, children, and adolescents [23,24].

Known causes of elevated troponin and B-type natriuretic peptide values are presented in Table 3 [20,23,25].

Here, we review the current analytical and clinical limitations for the cardiac troponins and natriuretic peptides in the pediatric population. It is intended for physicians and laboratory professionals to be aware of these limitations when performing testing and interpreting test results.

## 2. Cardiac Biomarkers

Laboratory diagnostics is an important part of the decision-making process in clinical practice. The number of potential cardiac biomarkers has grown significantly in recent years and remains a key focus of ongoing research.

However, newer biomarkers have certain advantages but also some limitations, especially in pediatric cardiology due to insufficient data for reliable diagnostic, prognostic, and therapeutic implications [26]. Multimarker panels, omics-based analyses of DNA, RNA, proteins, and metabolites, and bioinformatics are becoming increasingly promising; however, more laboratory and clinical studies are required to determine their value in the diagnosis, prognosis, and monitoring of pediatric patients with cardiovascular diseases [27,28,29].

Cardiac biomarkers reflect different pathophysiological mechanisms that are involved in cardiovascular diseases and can be classified according to the pathophysiological mechanisms that contribute to the development of cardiovascular diseases into the following categories: myocardial stretch, myocyte injury, myocardial remodeling, and co-morbidities (inflammation, oxidative stress, renal dysfunction, and neurohormonal activation) [23,26,30].

The most commonly used cardiac biomarkers in clinical practice are the natriuretic peptides and cardiac troponins. The natriuretic peptides are used to identify myocadiac stress and ventricular strain, whereas troponins are used to detect myocyte injury [23,30].

### 2.1. Natriuretic Peptides

B-type natriuretic peptide (BNP) is a cardiac natriuretic hormone synthesized as a preprohormone (pre-proBNP), and proBNP is formed after the removal of the signal peptide. BNP and N-terminal pro-B-type natriuretic peptide (NT-proBNP) are produced by the cleavage of proBNP in response to pressure overload and ventricular wall stretch. The stimulus due to increased volume or pressure in the left and/or right ventricle is followed by intensive synthesis of the pre-proBNP peptide, from which proBNP and an amino acid residue are formed by the action of cardiac neutral endopeptidases [31]. BNP is produced in the circulation by the proteolytic action of corin and furin on proBNP, whereby an equimolar amount of inactive NT-proBNP is also produced. In circulation, BNP is normally broken down by the action of various enzymes. BNP is an active hormone, and NT-proBNP is a biologically inactive peptide [32]. Furthermore, during post-translational maturation of proBNP, glycosylation of the proBNP molecule is possible, and the degree of glycosylation is highly individual. As a result, a heterogeneous mixture of native and possibly glycosylated proBNP, BNP, and NT-proBNP peptides is present in the blood. The degree of glycosylation is highly individual, both in healthy subjects and patients with different heart diseases. Glycosylation residues in the region of the proBNP molecule close to the cleavage site inhibit the processing of proBNP by furin and corin. ProBNP in glycosylated and nonglycosylated forms is found in healthy individuals and patients with heart failure. BNP in blood exists as intact BNP 1–32 and various truncated fragments, making it unstable [31,33].

Despite BNP and NT-proBNP being produced in equimolar amounts, the serum concentration of NT-proBNP is higher than the concentration of BNP because NT-proBNP has slower clearance from the circulation. The plasma half-life of NT-proBNP (1–2 h) is longer than that of BNP (15–20 min), and NT-proBNP is more stable in vitro than BNP [34]. Because NT-proBNP is excreted primarily by the kidney, its concentration depends on renal function. Both BNP and NT-proBNP concentrations are influenced by age, sex, and underlying cardiac condition. Natriuretic peptide levels in healthy children rise during the first 4 days and decrease rapidly during the first few weeks of life, due to the transition from fetal to neonatal circulation. Serum natriuretic peptide concentration is higher in the first year of life and decreases during childhood to adult concentration as the child reaches puberty. Females have higher levels of natriuretic peptides than males of the same age. In addition, the presence of structural heart abnormality may have an impact on baseline levels of natriuretic peptides [30,35]. The influence of metabolic markers such as obesity or serum lipid level on natriuretic peptide value has not yet been fully evaluated [36].

The active and inactive forms of natriuretic peptide are secreted predominantly by ventricular myocytes due to mechanical or hormonal stimulation of cardiomyocytes. During the neonatal period, both cardiac and noncardiac factors such as lung maturation and renal development can affect volume distribution and hemodynamics, resulting in natriuretic peptide level changes. The exercise induces morphological and functional changes in the cardiovascular system in the context of cardiac adaptation to stress. If the natriuretic peptide level is elevated during exercise and normalized after a period of recovery, it could be interpreted as a physiological rise [6]. BNP and NT-proBNP levels are generally elevated in children with heart disease with alterations based on volume versus pressure loading conditions and based on systolic versus diastolic dysfunction. Literature data suggest that serial determination of BNP and NT-proBNP may be used to monitor established heart disease or pathological conditions with secondary cardiac manifestations but should not be used as a sole diagnostic test [35]. An additional application for natriuretic peptides is their use in monitoring cardiotoxicity in pediatric patients after cancer treatment [23]. Although natriuretic peptides are used in the diagnosis and monitoring of heart diseases (the most common diagnoses include myocarditis and dilated cardiomyopathy), their concentrations may rise in acute conditions like respiratory infections, acute inflammatory disease, or sepsis, even without underlying heart disease (Table 3). These pathologies induce volume overload and hemodynamic changes. As children have a large proportion of water in their body, they are more susceptible to changes, and elevated natriuretic peptide levels may indicate the heart’s compensatory response to acute conditions rather than a primary heart disease [37].

### 2.2. Troponins

Troponins are members of a complex of proteins that modulate the calcium-mediated interaction between actin and myosin within striated muscle cells. There are three subunits of the troponin complex: troponin C (TnC) is a calcium-binding subunit, troponin I (TnI) inhibits the interaction of actin with myosin, and troponin T (TnT) binds the troponin complex to tropomyosin. Although the function of troponin is the same in all striated muscles, there is tissue specificity of TnI and TnT. The cardiac-specific TnI and TnT isoforms have a unique N-terminal amino acid chain different from their skeletal muscle forms. Most troponin complexes are mainly bound to the myofibrils, although 6–8% of TnT and 2.8–4.1% of TnI is cytosolic. There is an early release of cytosolic troponins after ischemic injury, followed by a prolonged release of bound troponins. As TnT has a big cytosolic pool, the release follows a biphasic pattern, while TnI has a smaller cytosolic pool, and the release is monophasic. Concentrations of both troponins begin to rise in the 4–8 h following injury and peak at 12–24 h. While TnI concentration may remain raised for 5–7 days, the concentration of TnT may remain raised for more than two weeks [38].

Troponin level rises during the first few days of life in healthy term and preterm neonates, peaking on day three, likely due to transient hypoxia at birth. Troponin concentration was significantly higher after delivery by cesarean section compared with vaginal delivery. Healthy termed neonates have a lower concentration compared with premature neonates. Serum troponin concentrations are higher in the first year of life, likely due to heart muscle development in early life, and gradually decrease to adult concentrations by adolescence. Minor gender-specific differences have been observed in adolescents, with higher concentrations in boys likely due to the greater cardiac mass in males. Children with decompensated congenital heart disease have a higher baseline troponin level than healthy children, regardless of heart failure status. Troponin values are generally higher in the pediatric population compared with those observed in healthy adult subjects [35,39,40].

Cardiac troponins are released during the process of myocyte necrosis, including an ischemic or hypoxic event. Although they are the most specific markers of myocyte injury, troponins are not causes or clinical-condition-specific biomarkers. Literature data suggest the clinical value of troponin measurement in diverse cardiac conditions in neonates, children, and adolescents.

Myocardial damage is rare in previously healthy children. While chest pain is a common symptom in children and adolescents, it is rarely of cardiac origin (1–3%). When elevated troponin levels are detected in these patients, the most frequent cause is myocarditis or pericarditis (approximately 50% of cases), followed by drug-induced vasospasm (most commonly cannabis), cardiac contusion, sepsis, and anomalous coronary artery outflow [41,42]. Although these conditions are uncommon, troponin measurement is increasingly becoming a standard part of the evaluation for children with cardiorespiratory symptoms admitted to intensive care units [43].

Children with congenital heart disease, whether symptomatic or asymptomatic, may have troponin levels that range from normal to elevated. Pressure-overloaded defects tend to cause higher troponin levels compared with volume-overloaded defects. Elevated troponin levels are commonly seen after heart surgery and are closely related to the amount of heart damage [24,35,39]. The release of troponin in the postoperative period is related not only to myocardial cell injury but also to surgery duration, techniques, and unavoidable surgical trauma. The determination of troponin level trends over postoperative time is important for detecting postoperative complications [35]. In addition, reduced renal clearance and continuous myocardial damage from exposure to kidney noncleared toxins may be associated with increased serum troponin levels in patients with chronic kidney disease [30]. The troponin level transitory variations after physical exercise may be caused by exercise-related myocyte micro-injuries and rapid early release of cytosolic troponins. The phenomenon of troponin fluctuation is not suggestive of myocardial necrosis but rather a secondary mechanism such as microvascular ischemia or heart metabolic deficiency [4]. Cardiac troponins also have strong negative predictive values, since undetectable or low concentrations would exclude ischemic heart stress [30].

## 3. Discussion

Early detection and differential diagnosis of cardiovascular diseases in the pediatric population are pivotal for rapid and successful treatment. However, clinical manifestations of pediatric cardiovascular diseases vary widely from mild chest pain to cardiogenic shock, but the presentation is often nonspecific, such as increased fatigue, decreased exercise capacity, abdominal pain, gastrointestinal symptoms, or upper respiratory infection symptoms. Clinical presentation and outcomes of heart disease that manifest in infants can differ significantly from those observed in older children and adolescents. Neonates may be especially difficult to diagnose because of their inability to express symptoms, congenital heart defects affect 1% of live births, and there are leading causes of death in the first year of life. Apparently healthy children often report chest pain, particularly after physical exercise, but an underlying cardiac cause is identified in only 5% of cases. Furthermore, undiscovered preexisting congenital heart defects may become a trigger for adverse cardiovascular events in apparently healthy young athletes. In addition, preexisting cardiovascular disease may increase the risk of cancer-therapy-related cardiotoxicity or may influence the severity and mortality of another disease [4].

Cardiac biomarkers can be used as an adjunctive marker in the evaluation and monitoring of pediatric patients. To date, more than 5000 original studies and review articles about children’s cardiac biomarkers have been published. However, currently, there are no validated guidelines or recommendations for how to interpret cardiac biomarkers in the pediatric population. Despite numerous literature data, the clinical application and interpretation of cardiac biomarkers in children have not been fully clarified. The results from the studies on reference values, as well as results from clinical studies, are difficult to compare with identical studies due to subject characteristics (gestational and chronological age, sex, pubertal status, menstrual cycle, exercise), assay characteristics (type of assay, generation of assay, analytical platform used), and experimental protocol characteristics (prospective or retrospective studies, reference population selection, patient population selection, inclusion and exclusion criteria, number of subjects).

The volume of blood samples required for cardiac biomarkers measurement is often an important limitation, especially in preterm neonates or low birth weight infants. Therefore, some reference interval studies included samples collected from subjects who had blood taken for other clinical indications. However, it is possible that these noncardiac conditions influence cardiac biomarker concentration [44]. The other studies reported reference intervals using blood samples from subjects who did not have any suspected clinical conditions, such as, for example, the CALIPER cohort or the LIFE child cohort [36,45]. So far, all reference value studies in the pediatric population have sampling errors, as they are conducted on a small number of study participants or do not encompass all pediatric age groups. Unfortunately, even larger studies do not satisfy the criteria of at least 300 cases for different age and sex groups to calculate the reference intervals or 99th percentile cut-offs [36,45,46,47].

While it is useful to establish reference intervals for natriuretic peptides and troponins in healthy children based on age and sex, we must consider the impact of congenital heart disease on baseline levels of natriuretic peptides and troponins. This is because the presence of structural cardiac abnormality impacts baseline biomarker levels, and therefore, the healthy children reference intervals cannot be used for similarly aged children with a structural abnormality [30]. In addition to the specific above-mentioned reference intervals, frequent exercise also affects reference values. Exercise-related myocardial and vascular adaptation processes are physiological processes and have an impact on baseline levels of cardio biomarkers. Therefore, the use of the general population reference intervals might not be appropriate for young athletes [4,48].

In healthy adults, the index of individuality is moderate for troponin, varying from 0.2 to 1.4, and low for NT-proBNP, varying from 0.5 to 0.64, depending on study methodology. These facts may suggest that reference intervals for troponin and NT-proBNP have limited clinical utility, while serial determinations may be more useful in making clinical decisions and therapeutic monitoring [47]. In adults, the change of 130% for BNP and 90% for NT-proBNP is necessary before the results of serial determinations can be considered statistically significant [49]. However, intra-individual variability of troponin and natriuretic peptides has not yet been studied in a pediatric population.

Early detectability of the troponin increase in blood depends on the analytical sensitivity of the specific troponin assay used. The International Federation of Clinical Chemistry and the American Association for Clinical Chemistry have assigned the term high sensitivity (hs) to troponin assays that have a coefficient of variation of ≤10% at the 99th percentile value and ≥50% of the detectable values above the limit of detection in a healthy population of both sexes [46]. This analytical sensitivity ranges from 1 to 3 ng/L. Most of these assays have been developed to measure levels of hsTnI, but only Roche has developed an hsTnT assay. The analytical performance of troponin methods has progressively improved during the last ten years, significantly affecting the diagnostic accuracy of troponin assays [50]. However, many articles do not clearly report the analytical characteristics of troponin methods used. In addition, there are multiple troponin assays and analytic platforms available in the marketplace, and one cannot extrapolate the results from one assay to another. The troponin assays vary widely in the specific troponin forms measured, the troponin fragments measured, the troponin epitopes, specific antibodies used, analytical platforms, sample types, minimum sample volume required, and overall diagnostic performance [39]. At present, measuring troponin in the pediatric population is still controversial. Some authors believe that troponin measuring can be useful in screening, diagnosis, and evaluation of pediatric patients with heart disorders, especially in the presence of clinical suspicion or an abnormal electrocardiogram. However, other authors believe that troponin measuring may provide minimal benefit while increasing costs. There is no universal standard for troponin monitoring, and laboratories use both conventional troponin assays and hs-troponin assays, limiting data comparison across literature data [39,51]. In addition, point-of-care-testing assays can measure troponin using only a drop of whole blood or plasma, which is a desirable sample, especially in preterm neonates or low-birth-weight infants. However, point-of-care-testing methods must have quality specifications equivalent to standard hs-troponin assays [39,52].

The heterogeneity of cardiovascular disease forms, the diversity of circulating forms of natriuretic peptides, the distinct expression of these forms in particular patients, and the difference between samples and immunoassays make the interpretation of natriuretic peptides complex and demanding.

Immunoassays used to measure BNP and NT-proBNP may cross-react with proBNP, and their accuracy depends on the assay and peptide form [31,33].

Similarly, NT-proBNP is highly glycosylated, which can interfere with immunoassay detection. Second-generation NT-proBNP assays target central regions of the molecule, but glycosylation may hinder antibody recognition. Deglycosylation of samples is recommended to improve accuracy, as glycosylation linked to other diseases can also affect NT-proBNP measurement [31,33].

BNP’s instability in blood results from its susceptibility to enzymatic proteolysis, and protease inhibitors can improve the accuracy of determination during sample collection. Also, immunoassays targeting specific regions of BNP may miss truncated forms, depending on the antibodies used [31].

## 4. Conclusions

Cardiac biomarkers can support the diagnosis, prognosis, and treatment of pediatric cardiovascular disease. However, their interpretation should always consider the patient’s medical history, clinical and laboratory findings, echocardiography, and other relevant information. Due to the lack of harmonization and standardization across immunoassays, as well as the absence of validated pediatric-specific guidelines, clinicians and laboratory staff should carefully evaluate the clinical and analytical characteristics of these biomarkers. Importantly, different specimen types and assay methods should not be used interchangeably for serial measurements, and the methodology employed should be clearly stated in the laboratory report.

## 5. Future Directions

Further studies are needed to define the 99th percentile concentration for all commercial hs-troponin assays and how they vary with age within each sex and ethnic origin.

In addition, further prospective cohort studies are needed to confirm the usefulness of screening and measuring troponin in the most common clinical conditions using standardized testing algorithms with a sufficient number of patients.

Also, future studies need to establish evidence-based cut-offs for specific indications to optimize utilization and standardize the interpretation of cardiac biomarkers in neonates, children, and adolescents.

## Figures and Tables

**Table 1:** Key insights into pediatric heart diseases.

Pediatric Heart Diseases	Key Insights	References
Congenital Heart Defects (CHD)	CHD is characterized by congenital malformation of heart walls, valves, and blood vessels. CHD causes 30% of fetal deaths, affects 1% of live births and is a leading cause of infant mortality. Causes: genetic mutations and epigenetic factors contribute to structural malformations, with 400 genes identified in CHD pathogenesis. CHD requires lifelong management due to its impact on morbidity and hospitalizations.	[8,9,10]
Cardiomyopathies	Hypertrophic cardiomyopathy (HCM): A genetic disorder causing heart muscle thickening and diastolic dysfunction. It is a leading cause of sudden cardiac death in young athletes. Dilated cardiomyopathy (DCM): Characterized by ventricular enlargement and systolic dysfunction, often caused by secondary heart diseases. Genetic overlap exists between HCM and DCM. Acquired cardiomyopathies may result from infections or cancer therapy.	[11,12]
Inflammatory Myocardial Diseases	Myocarditis: Inflammation of the heart muscle, often viral, leading to myocardial fibrosis and potentially chronic cardiomyopathy. Pericarditis: Inflammation of pericardial layers, primarily due to trauma or surgery, often presenting with chest pain. Myopericarditis and perimyocarditis represent overlapping syndromes.	[13,14,15,16]
Cardiotoxicity in Cancer Survivors	Advances in pediatric oncology have improved survival rates, but cancer therapies (chemotherapy and radiotherapy) increase the risk of heart failure and cardiomyopathy. Cardiotoxicity ranges from transient effects to progressive chronic conditions, driven by genetic, epigenetic, and environmental interactions.	[6,7]
Emerging Concerns	COVID-19 can cause myocarditis, pericarditis, and arrhythmias in children, especially in those with preexisting heart conditions. mRNA COVID-19 vaccines are associated with rare cases of immune-mediated myocarditis.	[5,6]

**Table 2:** Characteristics of cardiac biomarkers used in cardiology.

		Earliest Increase (Hours)	Achieving the Highest Value (Hours)	Time Period of Increased Values	Specificity % (95% CI)	Sensitivity % (95% CI)
Cardiac Troponins (cTn) (*)	Troponin T	3–4	10–24	10–14 days	71.8 ^a^ (67.6–75.9)	98.9 ^a^ (96.4–100)
	Troponin I	4–6		4–7 days		
Creatine kinase (CK) (**)		4–8	24–36	36–48 h	68 **	95 **
CK-MB		3–4	12–24	48–72 h	100 (91–100) ^b^	87 (74–95) ^b^
Myoglobin		1–3	6–12	12–24 h	76 ^c^ (72–81)	60 ^c^ (52–68)
Lactate dehydrogenase (LD) (**)		10–12	24–72	8–14 days	70 **	82 **
Aspartate aminotransferase (AST) (**)		12–24	24–48	10–14 days	71 **	75 **
BNP		The half-life is 20 min			63 ^d^ (52–73)	95 ^d^ (93–96)
NT-proBNP		The half-life is 60–120 min			43 ^d^ (26–62)	99 ^d^ (97–100)

Legend: NT-proBNP: N-terminal pro-B-type natriuretic peptide; CK–MB: isoenzyme of CK specific from myocardium. (*) The current gold standard in the laboratory diagnosis of acute myocardial infarction according to criteria for myocardial injury where detection of an elevated cTn value above the 99th percentile upper reference limit is defined as myocardial injury. The injury is considered acute if there is a rise and/or fall of cTn values [18]; (**) LDH is used to distinguish between acute and subacute AMI in the late phase (when other cardiac markers have already returned to their baseline levels), and AST is no longer used for AMI diagnosis. No available data for 95% CI [18]. (a) The pooled sensitivity and specificity meta-analysis data reflected the values obtained for serial measurements (cut-off point—upper reference limit and delta one hour) in patients with an adjudicated diagnosis of acute myocardial infarction [19]; (b) the pooled multicenter study sensitivity and specificity data for CK-MB reflected the values for serial measurement (cut-off point—upper reference limit and delta four hours) in patients with adjudicated diagnosis of acute myocardial infarction according to the universal definition [20]; (c) the sensitivity and specificity of myoglobin reflected the values obtained from admission data measurement (cut-off point—upper reference limit and the median time from onset of acute coronary syndrome symptoms to admission was 3.0 h) in patients with acute coronary syndrome (ACS) symptoms. The final diagnosis (ACS/non-ACS) was based on the consensus of two cardiologists, all available clinical information, and the hospital discharge letter [21]; (d) the pooled data of the meta-analysis of sensitivity and specificity are reflected values obtained by measuring BNP and NT-proBNP (with optimal cut-off point of 100 ng/L for BNP and 300 pg/mL for NT-proBNP are proposed to “rule out” a diagnosis of heart failure) in patients with suspected acute heart failure in an acute setting. The reference standard was the diagnosis of heart failure by retrospective review or the final hospital diagnosis [22].

**Table 3:** Pathological conditions associated with elevated levels of troponin (Tn) and B-type natriuretic peptide (BNP) in blood.

Cardiac Causes	Increased Concentration in Blood	Non-Cardiac Causes	Increased Concentration in Blood
Acute coronary syndrome	Tn and BNP	Sepsis/septic shock	Tn and BNP
Myocardial infarction	Tn and BNP	Acute renal failure	Tn and BNP
Heart failure	Tn and BNP	Pulmonary hypertension	Tn and BNP
Myocarditis/pericarditis	Tn and BNP	Pulmonary embolism	Tn and BNP
Cardiomyopathy	Tn and BNP	Perinatal asphyxia	Tn
Congenital heart defects	Tn and BNP	Stroke/subarachnoid hemorrhage	Tn
Arrhythmias	Tn and BNP	Acute respiratory distress	BNP
Myocardial contusion	Tn	Hyperthyroidism	BNP
Cardiotoxic drugs	Tn and BNP	Sleep apnea	BNP

## Data Availability

No data were generated during this study, so a data-sharing statement is not applicable to this article.
